# The physiology of survival: Space

**DOI:** 10.1113/EP093299

**Published:** 2025-10-10

**Authors:** Damian M. Bailey, Angelique van Ombergen

**Affiliations:** ^1^ Neurovascular Research Laboratory, Faculty of Life Sciences and Education University of South Wales Glamorgan UK; ^2^ Bexorg, Inc. New Haven , CT USA; ^3^ Directorate of Human and Robotic Exploration Programmes European Space Agency Cologne Germany; ^4^ Translational Neurosciences University of Antwerp Antwerp Belgium

*Twenty years from now you will be more disappointed by the things that you didn't do than by the ones you did do. So, throw off the bowlines. Sail away from the safe harbor. Catch the trade winds in your sails. Explore. Dream. Discover*.

Mark Twain (1835–1910)

On 14 January, 2004, the United States announced an ambitious ‘Vision for Space Exploration’, promising that humankind would establish a permanent presence beyond Earth. What sounded then like the rhetoric of science fiction has, within two decades, become the blueprint for a new era of exploration – science fact. Artemis is preparing to return humans to the Moon and, ultimately, to carry them further still – to Mars. A mission to the Red Planet – lying anywhere between ∼55 and 400 million km from Earth, depending on orbital alignment – would extend over a thousand days, subjecting crews to physiological and psychological stresses of unprecedented duration and severity, so daunting that many still consider the endeavour beyond human limits.

Space is, after all, unforgiving. Strip away the romance of rockets, the poetry of the pale blue dot, the cinematic allure of interstellar travel, and what remains is a vast vacuum filled with radiation, mind‐bending temperature swings and a gravitational void that slowly unravels biology, dismantling the very systems that keep us upright on Earth. The human body, exquisitely tuned to one atmosphere, one gravity and a narrow ecological niche, is ill‐suited to this alien environment. Surviving beyond Earth is not about grit alone – it is about physiology pushed to its absolute limits. And yet, it is precisely this hostility that presents physiologists with their most thrilling challenge in the greatest living laboratory ever conceived: to understand, predict and ultimately defend life against the cosmos itself.

Indeed, confronting adversity has long been a catalyst for progress, driving science and ingenuity in directions that comfort and safety would never have inspired. Physiology has taught us that environmental stress and human adaptation are inseparable. Altitude research has illuminated the limits of vascular oxygen transport, diving medicine has revealed the limits of pressure, and polar expeditions have revealed how human spirit and physiology entwine at the edge of endurance – the journeys across frozen frontiers were not only cartographic but existential, tracing the fragile line between survival and surrender. Space is now revealing how far human biology can bend without buckling. Where exploration was once defined by how far a ship could sail beyond the horizon, it is now measured by how far physiology can extend life in the most extreme and inhospitable environments. The question is no longer whether humans can survive in space, but how biology can be harnessed to make that survival possible, sustainable and perhaps even beneficial.

Central to this endeavour is recognising the cumulative totality of exposures astronauts face – the *space exposome* (Figure 1). More than 30 hazards are now catalogued by NASA's Human Research Program (NASA, 2023), ranging from cosmic radiation and altered gravity to circadian disruption, immune dysregulation, nutritional deficiency, and the psychological demands of isolation and confinement. Each hazard is life‐threatening, yet none exists in isolation – stressors interact and synergise, the whole becoming more than the sum of its parts, yielding a complex environment that challenges our physiology in novel and unpredictable ways (Bailey, 2025) (Figure 1).

**FIGURE 1:**
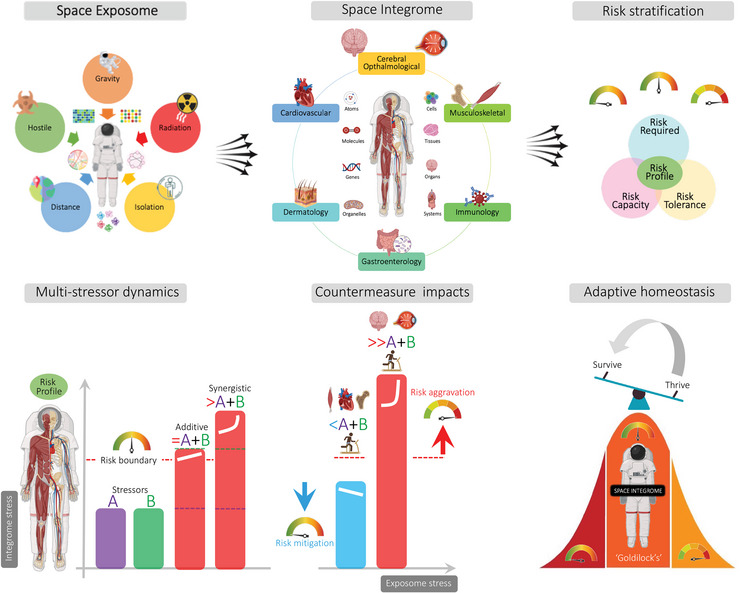
Survival in space – integrated phenotyping of the *space* *exposome–integrome* nexus. The *space exposome* captures the cumulative burden of environmental stressors encountered during spaceflight. The *space* *integrome* reframes this challenge – a systems‐level phenotype reflecting the interactions, synergies and antagonisms that emerge when these stressors converge upon human biology. Unlike the *exposome*, which can risk reductionist partitioning of effects, the *integrome* emphasises deep integration across scales – molecular, cellular, organ‐level and behavioural – exposing unanticipated crosstalk between pathways that shape overall risk profiles. The physiological response is non‐linear, resistant to single‐variable simulation, and often greater than the sum of its parts. Stressors may act independently in an additive manner (A + B) or synergistically in ways that amplify vulnerability (> A + B). Such dynamics underpin conditions including spaceflight‐associated neuro‐ocular syndrome, where vascular, intracranial and ocular physiology collide in an unpredictable fashion (>> A + B). Countermeasures are therefore less about neutralising single insults than about reshaping the integrated risk landscape and mitigate risk (< A + B) to an acceptable, albeit currently undefined boundary threshold (Goldilock's sweet spot). By accelerating discovery and validation of biomarkers across this *exposome–integrome* nexus, we can uncover novel pathways, generate fresh hypotheses and compress the timeline from mechanism to countermeasure. This integrative approach does more than map survival in space: it illuminates physiology as a networked enterprise, one that thrives on complexity rather than reductionism. Figure created with BioRender.com and adapted from (Bailey, 2025; Bailey et al., 2025), with permission (Wiley and Springer Nature).

The spacesuit is an astronaut's lifeline – a self‐contained biosphere and integrated life‐support system meticulously engineered to counter the lethal physics and unforgiving physiology of space. It provides pressure to prevent ebullism – the boiling of body fluids, including saliva and tears, in a vacuum – and provides precious oxygen that would otherwise lead to anoxia‐induced unconsciousness within 6–10 s (Bailey, 2019), while preserving a workable degree of mobility. Multiple layers of Mylar, Dacron and Kevlar or similar high‐strength fibres offer thermal insulation and protective armour against micrometeoroid impacts, while beneath them a liquid cooling and ventilation garment circulates water to dissipate metabolic heat, protecting astronauts from the violent thermal swings of space where temperatures can plummet to −150°C in shadow or soar to 120°C in direct sunlight (Neukart, 2024). A gold‐coated polycarbonate visor filters ultraviolet and infrared radiation, protecting the retina from catastrophic burns, and embedded sensors continuously monitor the partial pressures of oxygen and carbon dioxide, humidity and core body temperature, feeding data to the backpack life‐support unit that sustains gas exchange and thermal balance.

The current NASA Extravehicular Mobility Unit (EMU), first used in 1983 on Space Shuttle missions and continually adapted for International Space Station (ISS) operations sustains this delicate balance at 222 mmHg and 100% oxygen, a pressure far below the station's cabin maintained at 760 mmHg and 21% oxygen, necessitating pre‐breathe protocols to flush nitrogen and mitigate decompression sickness (DCS) (Kluis & Diaz‐Artiles, 2021). Next‐generation suits built for planetary exploration, including NASA's xEMU and commercial EVA systems, are being developed to operate at higher pressures (up to 424 mmHg and 34% oxygen, optimising trade‐offs between DCS, hypoxia, flammability and several other factors; Norcross et al., 2012), thereby reducing pre‐breathe requirements and improving safety, though at the cost of increased stiffness and reduced mobility. Every design decision reflects a physiological trade‐off: too little pressure and circulatory survival collapses in seconds; too much, and the astronaut becomes locked in a rigid shell.

But to truly understand the hostility of space is to recognise that the hazards are not only threats – they are also stimuli, capable of provoking adaptations that have the potential to unlock the adaptive ‘power of physiology’ (Bailey, 2022). Radiation, for example, is the most intractable enemy of long‐duration missions. Galactic cosmic rays, composed of high‐energy protons and heavy ions, induce complex clustered DNA lesions that overwhelm repair pathways, increasing the likelihood of chromosomal instability, mutagenesis and ultimately oncogenesis (Furukawa et al., 2020; Li et al., 2018; Kern et al., 2025). However, ongoing translational research is identifying biomarkers of susceptibility, delineating repair mechanisms, and developing protective strategies that not only mitigate radiation injury in astronauts but also hold promise for advancing cancer therapeutics and radioprotective interventions on Earth (Akbarialiabad et al., 2025). Microgravity, too, has a Janus‐face – on one side driving bone loss and muscle atrophy, while on the other offering a powerful model of accelerated ageing that allows scientists to probe mechanisms and trial countermeasures at a speed unattainable in terrestrial settings (Mozalbat et al., 2025). Spaceflight‐associated neuro‐ocular syndrome (SANS) has illuminated entirely new aspects of fluid dynamics, intracranial pressure and ocular physiology (Joe, 2024). Each hazard transforms adversity into an opportunity for discovery.

It is within the *space integrome* – the body's seamless, integrated response to these exposures – that the true story of resilience and survival unfolds (Bailey, 2025). Physiology is not simply a collection of isolated systems but an orchestra of interactions, where survival depends on the harmony of countless finely tuned processes working in concert (Lemoine & Pradeu, 2018). Bone resorption alters calcium balance and renal function. Fluid shifts impact both cerebral perfusion and cardiac preload. Immune suppression intersects with wound healing, haematopoiesis and viral reactivation. By studying this functional interconnectedness under the stresses of spaceflight, we are learning more about the nature of integration itself. Human biology, far from fragile, displays a remarkable ability to adapt, recalibrate and establish new equilibria in alien environments – a resilience that forms the foundation of future exploration.

Countermeasures form the scaffolding of human adaptability, and here the story is one of steady optimism. Skylab crews in the 1970s improvised resistance training with rudimentary devices, establishing the foundation for today's sophisticated regimens. Cosmonauts on Mir endured prolonged isolation yet showed that, with discipline, humans could live and work in orbit for more than a year. Apollo astronauts, operating in a far more primitive environment, demonstrated that the body could withstand rapid transitions from Earth gravity to lunar gravity and back within little more than a week. Each successive mission has stretched the boundaries of possibility and reaffirmed that human physiology is less brittle than once feared.

In modern times, astronauts aboard the ISS now dedicate up to 2.5 h (including preparation) each day to structured exercise – resistive training, aerobic intervals and treadmill running with harnesses (Petersen et al., 2016; Scott et al., 2023). Countermeasures have become among the most advanced applications of physiology, offering meaningful, albeit partial protection against the exposome's cumulative toll. Muscle strength can be preserved to a degree, bone loss attenuated and cardio/cerebrovascular decline moderated, though none are fully prevented (Fernandez‐Gonzalo et al., 2025). Pharmacological approaches, from bisphosphonates (Rosenthal et al., 2024) to antioxidants (Gomez et al., 2021), are advancing through trials, while nutritional strategies now emphasise tailored diets that stabilise metabolism and glycaemic control (Barbero Barcenilla et al., 2025). Emerging technologies, including lower‐body negative pressure (Harris et al., 2020) and centrifuge‐based artificial gravity (Clement et al., 2015), are being reimagined in more practical forms, expanding the range of tools available. No single intervention is sufficient, yet together these approaches mark steady progress – an evolving countermeasure portfolio that strengthens with each mission and incrementally closes the gap between survival and sustainability.

The translation of this science into ‘lived’ experience is embodied most clearly by the astronauts themselves. When Scott Kelly spent 340 consecutive days aboard the ISS in 2015–2016 – the longest continuous mission by a NASA astronaut – his body became a living dataset, anchoring the landmark NASA ‘Twins Study’ that revealed how prolonged spaceflight reshapes human biology in ways both transient and enduring. Compared with his twin brother, Mark, who remained on Earth, Scott revealed a host of adaptations: alterations in telomere length, changes in gene expression, shifts in immune function, and altered cognition (Garrett‐Bakelman et al., 2019). Many of these changes reverted after return to Earth, underscoring the remarkable plasticity of human biology. Peggy Whitson, who spent more than 665 days in space across three missions, provides another example. In her late fifties – an age at which most would expect diminished physiological resilience – she maintained physical and cognitive performance at levels equal to, or surpassing, those of younger colleagues. Her experience is a testament not only to individual determination but to the robustness of countermeasures and the adaptability of female physiology, which we still know vanishingly little about.

What makes the current moment particularly exciting is the convergence of physiology with the burgeoning digital revolution. Biosensors now allow continuous monitoring of ‘vitals’, ranging from heart rate variability to intracranial dynamics, and biomarkers in hair, skin, sweat, saliva, urine and blood. Multi‐omics are providing a fingerprint of human adaptation in real time (Overbey et al., 2024). These data pipelines can feed into digital twins (Katsoulakis et al., 2024) – virtual models of an astronaut's physiology that update dynamically, predict risks and test countermeasures before they are applied. The astronaut of the near future will not be a passive passenger subject to unknown risks but an active participant in their own physiological stewardship. The *integrome* will be mapped, modelled and managed with unprecedented physiological precision (Bailey, 2025).

There is an increasing recognition that adversity itself can be harnessed as a positive and protective force. The principle of hormesis – the adaptive, beneficial response to low‐level stressors – has long informed research in toxicology, exercise physiology and ageing, demonstrating that controlled exposure to stress can enhance resilience, stimulate repair mechanisms and improve overall resilience (Wan et al., 2024). Space now offers a new domain for its application. Could carefully modulated exposures to hypoxia, microgravity or radiation induce protective adaptations that exceed those available on Earth? Could intermittent microgravity, interspersed with controlled hypoxia, not only preserve cerebrovascular and musculoskeletal integrity but also enhance metabolic flexibility? Such questions signal a shift from a purely defensive approach to one that seeks opportunity: viewing space not merely as an environment to endure, but as a milieu in which adaptive resilience can be stimulated and optimised.

The dividends extend far beyond astronauts. Osteoporosis therapies draw directly on bone loss research in orbit. Insights into sarcopenia, immune ageing, cardiovascular deconditioning and circadian disruption are already shaping terrestrial medicine. The lessons of SANS may prove transformative in understanding idiopathic intracranial hypertension. Mental health research in the context of isolation and confinement is offering new tools for resilience and wellbeing on Earth – lessons made all the more relevant in relation to the COVID‐19 pandemic, when billions confronted prolonged isolation, disrupted routines and psychological strain reminiscent of life in space (Chouker & Stahn, 2020). In this sense, astronauts serve not just as explorers but as accelerated models of human ageing and adaptation. Every countermeasure becomes a potential therapy for patients on Earth (Shelhamer et al., 2020).

Nor should we underestimate the cultural and inspirational value of surviving in space. Human exploration has always been about more than experimentation or data collection; it is about redefining what it means to be human. The Apollo missions captured global imagination not merely because astronauts set foot on the Moon, but because they revealed both the fragility and resilience of life against a vast and hostile cosmos. Today, the physiology of space carries the same narrative power: biology can adapt in extraordinary ways, and survival beyond Earth is not only possible – it is the foundation for thriving in environments far removed from our planetary origin. Risk, of course, will never disappear. The Apollo 1 fire, *Challenger* and *Columbia* disasters, and countless near misses remind us that pushing boundaries carries cost. Yet risk is not synonymous with failure. Physiology teaches us that stress and adaptation are inseparable – two sides of the same coin – and it is within this dynamic interplay that progress and resilience emerge. The very pressures that threaten survival also drive the innovations that make it achievable. Astronauts of the future will not be immune to hazard, but they will be equipped with the knowledge, tools and resilience to meet it, and it is within this balance of risk and adaptation that the essence of exploration lies.

Humanity's push towards Mars will not be realised because it is easy, but precisely because it is hard – and because biology, when deeply understood and carefully supported, reveals capacities more remarkable than we ever imagined. In the physiology of survival lies the physiology of possibility. By studying the body at its limits, we not only make exploration viable; we uncover the very essence of human resilience and expand the horizon of what it means to endure, adapt, survive and thrive.

## AUTHOR CONTRIBUTIONS

*Conceiving the idea and writing the first draft of the manuscript*: Damian M. Bailey. *Editing and revising the manuscript*: Damian M. Bailey and Angelique van Ombergen. All authors have read and approved the final version of this manuscript and agree to be accountable for all aspects of the work in ensuring that questions related to the accuracy or integrity of any part of the work are appropriately investigated and resolved. All persons designated as authors qualify for authorship, and all those who qualify for authorship are listed.

## CONFLICT OF INTEREST

D.M.B. is Editor‐in‐Chief of *Experimental Physiology* and outgoing Chair of the Life Sciences Working Group and outgoing member of the Human Spaceflight and Exploration Science Advisory Committee to ESA. D.M.B. is a current member of the ESA‐HRE‐Biology Panel and Space Exploration Advisory Committees to the UK and Swedish National Space Agencies. A.v.O. is ESA's Chief Exploration Scientist, Directorate of Human and Robotic Exploration to ESA, Associate Editor of NPJ Microgravity and Visiting Professor at the University of Antwerp, Belgium.

## FUNDING INFORMATION

D.M.B. is supported by a Royal Society Wolfson Research Fellowship (Grant No. WM170007).
